# Hydrogen peroxide poisoning: an unusual cause of portal venous gas

**DOI:** 10.1259/bjrcr.20150283

**Published:** 2016-01-19

**Authors:** Evyn Arnfield, Hemant Bhardwaj, Nicholas Brown, Michael Handy, Perry Cleland

**Affiliations:** ^1^ Department of Medical Imaging, Royal Brisbane and Women’s Hospital, Herston, QLD, Australia; ^2^ Department of Medical Imaging, Wesley Hospital, Auchenflower, QLD, Australia

## Abstract

Hydrogen peroxide (H_2_O_2_) is an oxidizing agent found in many household products. It is a clear liquid at room temperature, allowing it to be mistaken for water if unlabelled. Ingestion of H_2_O_2_ can have serious sequelae even in low volumes and concentrations.We present a case study of H_2_O_2_ poisoning and discuss the pertinent radiographical findings.

## Summary

Hydrogen peroxide (H_2_O_2_) is an oxidizing agent found in many household products. It is a clear liquid at room temperature, allowing it to be mistaken for water if unlabelled. Ingestion of H_2_O_2_ can have serious sequelae even in low volumes and concentrations. We present a case study of H_2_O_2_ poisoning and discuss the pertinent radiographical findings.

## Clinical presentation

A healthy 21-year-old male unintentionally ingested approximately one mouthful of 3% H_2_O_2_ stored in his relative’s refrigerator. He presented to the emergency department with vomiting and pain in his mouth, throat and epigastrium. He was tachycardic (100 beats min^−1^) and mildly hypertensive (155/100 mmHg) but not hypoxic (SpO_2_ 97% on room air). A clinical examination revealed dysphonia with mild erythema and oedema of the oropharynx and uvula; the abdominal and respiratory examinations were otherwise unremarkable. Blood and biochemical investigations were also normal.

## Imaging findings

**Figure 1.**
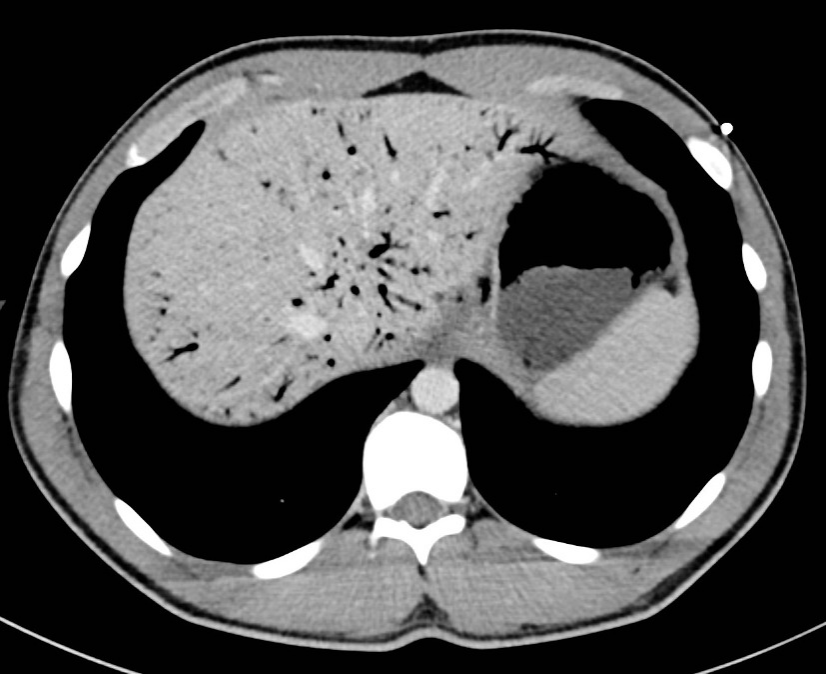
Axial portal venous phase CT image showing gastric mucosal thickening with portal venous gas.

**Figure 2.**
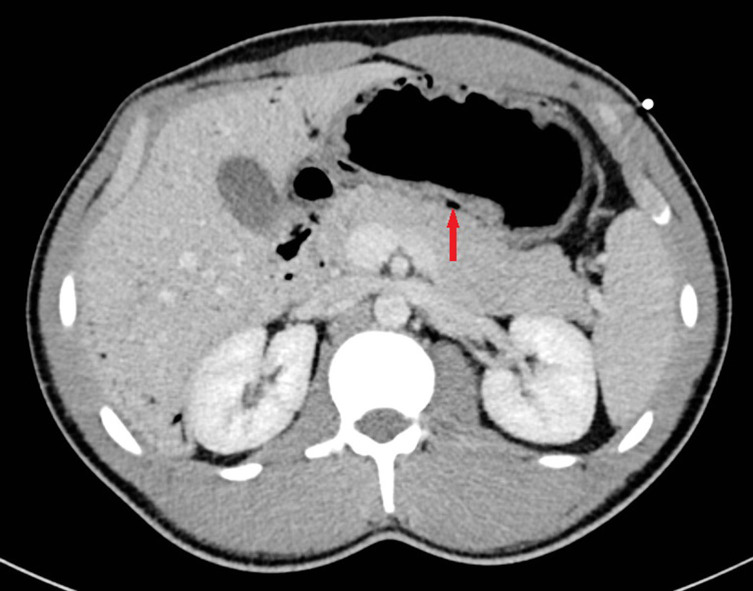
Axial portal venous phase CT image showing gastric mucosal thickening and pneumatosis (arrow).

**Figure 3.**
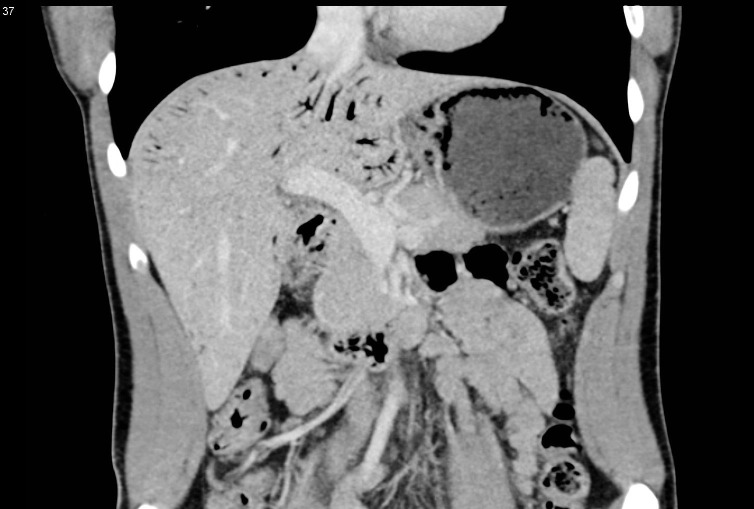
Coronal portal venous phase CT image showing gastric mucosal thickening and pneumatosis with portal venous gas.

A CT scan was performed to exclude gastrointestinal perforation, which demonstrated pneumatosis and mucosal thickening throughout the stomach and proximal duodenum, as well as extensive portal venous gas (Figures 1–3).

## Treatment

The patient was intubated owing to concerns for developing airway involvement and gastrointestinal perforation, and conservative treatment with intravenous piperacillin/tazobactam 4.5 g four times daily and intravenous pantoprazole infusion at 8 mg hour^−1^ was commenced prior to transfer to a tertiary intensive care unit.

## Outcome

**Figure 4.**
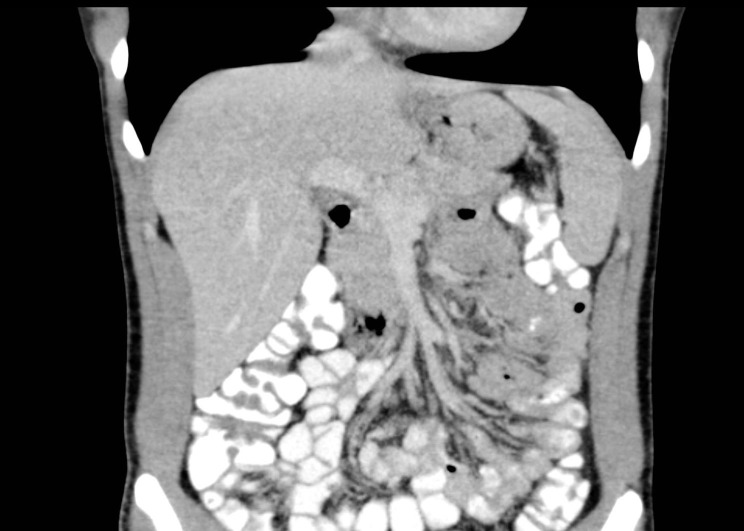
Coronal CT image with intravenous and oral contrast showing interval resolution of pneumatosis and portal venous gas.

An upper endoscopy performed 3 days after the ingestion was normal, with no evidence of mucosal injury. A repeat CT scan at this time revealed interval partial resolution of the bowel wall thickening and complete resolution of the pneumatosis and portal venous gas (Figure 4). The patient was discharged 3 days after presentation on oral pantoprazole and antibiotic therapy, and made a full recovery.

## Discussion

H_2_O_2 _is an oxidizing agent that is available in concentrations ranging from 3% to 90%.^1^ It is found in numerous household products, including disinfectants, hair dyes, bleaches and stain removers. It is a clear, colourless liquid at room temperature,^2^ allowing it to be mistaken for water if unlabelled, as in this case. Recently, H_2_O_2_ has also been sold for consumption in small volumes following promotion of its purported natural health benefits,^3^ despite multiple documented fatalities from its ingestion^2^ and no evidence to demonstrate health benefits of any kind. Upon contact with the enzyme catalase in the gastric mucosa, H_2_O_2_ undergoes rapid decomposition into oxygen and water (2H_2_O_2 _→ 2H_2_O + O_2_ + heat).^4^ If the amount of oxygen liberated exceeds the maximum solubility of blood, bubbles migrate through the epithelial interstices and gas embolism may occur, manifesting as pneumatosis^3^ or gas within the portal venous system,^5^ brain^6^ and coronary arteries.^7^ Toxicity is also caused by direct caustic injury to the gastric mucosa, resulting in gastritis and potential rupture, as well as cytotoxicity from lipid peroxidation.^1^ This particular case is unusual because gas embolism usually only occurs with ingestion of the stronger 35% H_2_O_2_ solution, with only a few other published case reports of portal venous gas following ingestion of the 3% solution.^8,9^

## Learning points

1. Ingestion of H_2_O_2_, a common household item, can have serious sequelae even in low volumes and concentrations.
2. Radiographical findings may include gastritis, pneumatosis, perforation and portal venous gas.
